# Supplementary material for: The sensitivity of simulated streamflow to individual hydrologic processes across North America
Source: Nat Commun. 2022 Jan 24;13:455. doi: 10.1038/s41467-022-28010-7 (PMC8786896; doi:10.1038/s41467-022-28010-7)
Supplement: Supplementary file 1 — Supplementary Information [file 41467_2022_28010_MOESM1_ESM.pdf]

The Sensitivity of Hydrologic Model Parameters and Processes Regarding Simulated Streamflow Across North America

Juliane Mai,* James R. Craig, and Bryan A. Tolson
Dept. of Civil and Environmental Engineering, University of Waterloo, Waterloo, ON, Canada.

Richard Arsenault
Dept. of Construction Engineering, École de technologie supérieure, Montreal, QC, Canada.
(Dated: November 26, 2021)

SUPPLEMENTARY MATERIAL

Details about the Blended Model

The Blended Model is a lumped hydrologic model defined within the Raven hydrologic modeling framework [1]. The Blended Model uses the weighted average of several chosen process implementations for key processes to simulate streamflow instead of using one single parametrization per process.

As an example, a (simplified) blended model could be defined as:

$$f_{shared}(x,w) = (w_{d1}D_1 + w_{d2}D_2) \cdot (w_{e1}E_1 + w_{e2}E_2 + w_{e3}E_3) + (w_{f1}F_1 + w_{f2}F_2) \tag{S1}$$

with

$$w_{d1} + w_{d2} = 1 \tag{S2}$$

$$w_{e1} + w_{e2} + w_{e3} = 1 \tag{S3}$$

$$w_{f1} + w_{f2} = 1 \tag{S4}$$

where D_1 and D_2 could be, for instance, be two options for one process. For example, deriving infiltration could be performed once using the infiltration definition of HMETS (D_1) and once derived as defined in the HBV model (D_2). The infiltration outputs D_1 and D_2 are then weighted using w_{d1} and w_{d2} to derive the infiltration estimate Raven will use for the remainder of the simulation. The overall flowchart of the model, given all hydrologic processes involved, is given in the flowchart in Fig. S1. A detailed description of all processes and process options can be found in the Supplementary Material of Mai *et al.* [2] and in the Raven documentation [1].

The Blended Model as introduced by Mai *et al.* [2] and used by Chlumsky *et al.* [3] uses three different options M_i for the infiltration process, three options N_i for quickflow, two options O_i for evaporation, two options P_i for baseflow, and three options Q_i for snow balance. All other processes, i.e., convolution of surface runoff R_1 and delayed runoff S_1, potential melt T_1 , percolation U_1 , rain–snow partitioning V_1 , and precipitation correction W_1, are used with one fixed process option. The remaining processes also have only one option, but none of them contains tunable parameters. They are merged to a "remaining" process X_1, which will never appear in the sensitivity analysis because it is constant. When the first option of each of the processes M_1, N_1, O_1, P_1, Q_1, R_1, S_1, T_1, U_1, V_1, W_1, and X_1, is chosen and parameter x_{35} is set to zero, the Raven setting emulates the HMETS model [4] perfectly. All other combinations are unnamed models.

Details of process options and parameters can be found in Appendix C (Tables C1 and C2) in Mai *et al.* [2] and are added to the Supplementary Material herein (Table S1 and Table S2) for the convenience of readers.

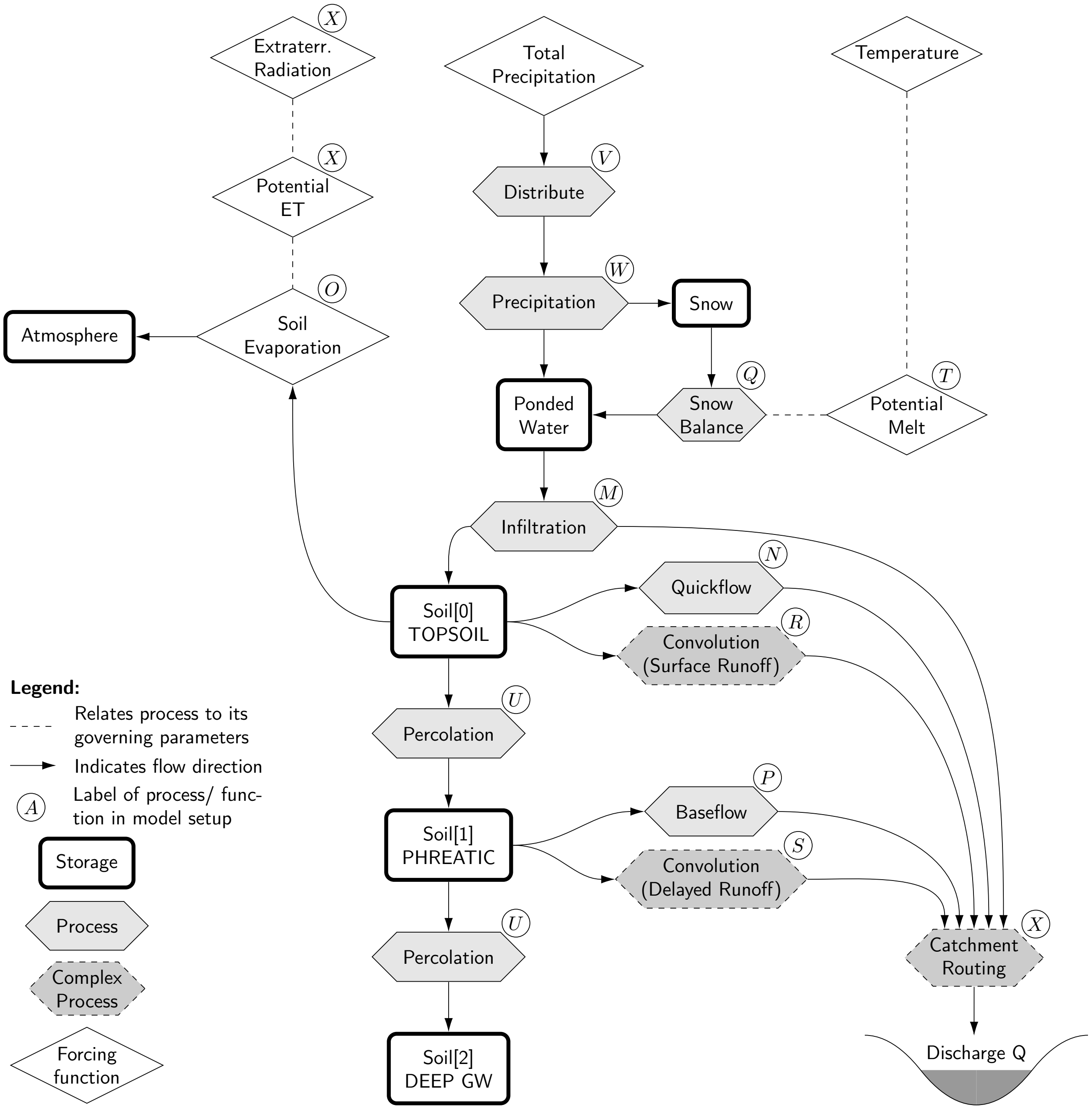

FIG. S1. **Flowchart Blended Model.** The model schematic of the model structure used in this study. The connection of storages (boxes with thick outlines), processes (hexagonal shapes), and forcing functions (diamond shapes) are shown. Some processes are simplified in this schematic (hexagonal shape with dashed outline). The labels used for the processes/functions in this study are indicated by the circled letters to the right of the processes and forcing functions. The five processes M to Q are used here with multiple options, while the processes R to W are fixed, with only one option. The processes labeled X are the ones that are fixed, with one option, and this option does not contain tunable parameters. Hence, the sensitivity of the processes X is already prior known to be zero. The processes and options, as well as the parameters active in each option, are listed in Table S1 and Table S2, respectively. This figure is taken from Mai *et al.* [2] (Supplements Figure S1 therein).

TABLE S1. **Processes and process options used for the Raven setup.** In total, 3×3×2×2×3×1×1×1×1×1×1×1 = 108 models are possible. The first option of each process M_1, N_1, O_1, P_1, Q_1, R_1, S_1, T_1, U_1, V_1, W_1, and X_1 resembles the HMETS model if parameter x_{35} is set to zero. All other combinations are artificial models. All process options, however, are used in different hydrologic models. The model parameters active in each option are listed as well. The ranges and a description of the parameters can be found in Table S2. This table is taken from Mai *et al.* [2] (Appendix C Table C1 therein).

Process	Process option		Active parameters
Processes with multiple options:			
Infiltration	M_1	INF_HMETS	$\{x_1, x_{29}\}$
"	M_2	INF_VIC_ARNO	$\{x_2, x_{29}\}$
"	M_3	INF_HBV	$\{x_3, x_{29}\}$
Quickflow	N_1	BASE_LINEAR_ANALYTIC	$\{x_4, x_{29}\}$
"	N_2	BASE_VIC	$\{x_5, x_6, x_{29}\}$
"	N_3	BASE_TOPMODEL	$\{x_5, x_6, x_7, x_{29}\}$
Soil evaporation	O_1	SOILEVAP_ALL	$\{x_8, x_{29}\}$
"	O_2	SOILEVAP_TOPMODEL	$\{x_8, x_9, x_{10}, x_{29}\}$
Baseflow	P_1	BASE_LINEAR_ANALYTIC	$\{x_{11}\}$
"	P_2	BASE_POWER_LAW	$\{x_{11}, x_{12}\}$
Snow balance	Q_1	SNOBAL_HMETS	$\{x_{13}, \ldots, x_{18}\}$
"	Q_2	SNOBAL_SIMPLE_MELT	–
"	Q_3	SNOBAL_HBV	$\{x_{18}, x_{19}\}$
Processes with single option:			
Convolution (surface runoff)	R_1	CONVOL_GAMMA	$\{x_{20}, x_{21}\}$
Convolution (delayed runoff)	S_1	CONVOL_GAMMA_2	$\{x_{22}, x_{23}\}$
Potential melt	T_1	POTMELT_HMETS	$\{x_{24}, x_{25}, x_{26}, x_{27}\}$
Percolation	U_1	PERC_LINEAR	$\{x_{28}, x_{29}, x_{30}, x_{35}\}$
Rain-snow partitioning	V_1	RAINSNOW_HBV	$\{x_{31}, x_{32}\}$
Precipitation correction	W_1	RAINSNOW_CORRECTION	$\{x_{33}, x_{34}\}$
Processes with single option but no tunable parameter combined together to create process X_1:			
Extraterr. Shortwave Gener.	X_1	SW_RAD_DEFAULT	–
Potential evapotranspiration	X_1	PET_OUDIN	–
In-catchment routing	X_1	ROUTE_DUMP	–
In-channel routing	X_1	ROUTE_NONE	–

TABLE S2: **The model parameters x_i used for the Raven setup.** The parameters are uniformly distributed in the range given. The process option shows where the corresponding parameter is active. The Raven table and parameter name can be used to locate the parameter in the Raven setup files. A three-layer soil model was used here, with the third (groundwater) layer being of infinite depth. The TOPSOIL is the upper soil layer while PHREATIC is the lower soil layer. The three Raven parameters, FIELD_CAPACITY TOPSOIL, SNOW_SWI_MAX, and MAX_MELT_FACTOR, are derived using a sampled parameter (x_{10}, x_{14}, and x_{25}) and SAT_WILT TOPSOIL, SNOW_SWI_MIN, and MIN_MELT_FACTOR, respectively, to make sure that one parameter is always larger than the other. The baseflow coefficients, BASEFLOW_COEFF TOPSOIL and PHREATIC, are derived from parameters x_4 and x_{11} to allow a logarithmic sampling. This table is taken from Mai *et al.* [2] (Appendix C Table C2 therein).

**Param.**	**Range**	**Unit**	**Proc. Opt.**	**Raven table**	**Parameter name**
Infiltration:					
x_1	$[0.0, 1.0]$	-	M_1	LandUseParameterList	HMETS_RUNOFF_COEFF
x_2	$[0.1, 3.0]$	-	M_2	SoilParameterList	B_EXP TOPSOIL
x_3	$[0.5, 3.0]$	-	M_3	SoilParameterList	HBV_BETA TOPSOIL
Quickflow:					
x_4	$[-5.0, -1.0]$	1/d	N_1	SoilParameterList	BASEFLOW_COEFF TOPSOIL = 10.0^{x_4}
x_5	$[0.0, 100.0]$	mm/d	N_2, N_3	SoilParameterList	MAX_BASEFLOW_RATE TOPSOIL
x_6	$[0.5, 2.0]$	-	N_2, N_3	SoilParameterList	BASEFLOW_N TOPSOIL
x_7	$[5.0, 10.0]$	m	N_3	TerrainClasses	TOPMODEL_LAMBDA
Evaporation:					
x_8	$[0.0, 3.0]$	-	O_1, O_2	SoilParameterList	PET_CORRECTION TOPSOIL
x_9	$[0.0, 0.05]$	frac	O_2	SoilParameterList	SAT_WILT TOPSOIL
x_{10}	$[0.0, 0.45]$	frac	O_2	SoilParameterList	FIELD_CAPACITY TOPSOIL = SAT_WILT TOPSOIL + x_{10}
Baseflow:					
x_{11}	$[-5.0, -2.0]$	1/d	P_1, P_2	SoilParameterList	BASEFLOW_COEFF PHREATIC = $10.0^{x_{11}}$
x_{12}	$[0.5, 2.0]$	-	P_2	SoilParameterList	BASEFLOW_N PHREATIC
Snow balance:					
x_{13}	$[0.0, 0.1]$	frac	Q_1	GlobalParameter	SNOW_SWI_MIN
x_{14}	$[0.01, 0.3]$	frac	Q_1	GlobalParameter	SNOW_SWI_MAX = SNOW_SWI_MIN + x_{14}
x_{15}	$[0.005, 0.1]$	1/mm	Q_1	GlobalParameter	SWI_REDUCT_COEFF
x_{16}	$[-5.0, 2.0]$	°C	Q_1	LandUseParameterList	DD_REFREEZE_TEMP
x_{17}	$[0.0, 1.0]$	-	Q_1	LandUseParameterList	REFREEZE_EXP
x_{18}	$[0.0, 5.0]$	mm/d/°C	Q_1, Q_3	LandUseParameterList	REFREEZE_FACTOR
x_{19}	$[0.0, 0.4]$	frac	Q_3	GlobalParameter	SNOW_SWI
Convolution (surface runoff):					
x_{20}	$[0.3, 20.0]$	-	R_1	LandUseParameterList	GAMMA_SHAPE
x_{21}	$[0.01, 5.0]$	-	R_1	LandUseParameterList	GAMMA_SCALE
Convolution (delayed runoff):					
x_{22}	$[0.5, 13.0]$	-	S_1	LandUseParameterList	GAMMA_SHAPE2
x_{23}	$[0.15, 1.5]$	-	S_1	LandUseParameterList	GAMMA_SCALE2
Potential melt:					
x_{24}	$[1.5, 3.0]$	mm/d/°C	T_1	LandUseParameterList	MIN_MELT_FACTOR
x_{25}	$[0.0, 5.0]$	mm/d/°C	T_1	LandUseParameterList	MAX_MELT_FACTOR = MIN_MELT_FACTOR + x_{25}
x_{26}	$[-1.0, 1.0]$	°C	T_1	LandUseParameterList	DD_MELT_TEMP
x_{27}	$[0.01, 0.2]$	1/mm	T_1	LandUseParameterList	DD_AGGRADATION
Percolation:					
x_{28}	$[0.00001, 0.02]$	1/d	U_1	SoilParameterList	PERC_COEFF TOPSOIL
x_{35}	$[0.0, 0.02]$	1/d	U_1	SoilParameterList	PERC_COEFF PHREATIC
Rain-snow partitioning:					
x_{31}	$[-3.0, 3.0]$	°C	V_1	GlobalParameter	RAINSNOW_TEMP
x_{32}	$[0.5, 4.0]$	°C	V_1	GlobalParameter	RAINSNOW_DELTA
Precipitation correction:					
x_{33}	$[0.8, 1.2]$	-	W_1	Gauge	RAINCORRECTION
x_{34}	$[0.8, 1.2]$	-	W_1	Gauge	SNOWCORRECTION
Soil model:					
x_{29}	$[0.0, 0.5]$	m	$M_{1,2,3}$,$N_{1,2,3}$ $O_{1,2,3,4}$, U_1	SoilProfiles	thickness TOPSOIL
x_{30}	$[0.0, 2.0]$	m	U_1	SoilProfiles	thickness PHREATIC

Comparison of xSSA Sensitivity Results With Previous Study

The sensitivity index estimates derived in this work are compared to a previous continental sensitivity study presented by Markstrom *et al.* [5]. In that study, the Fourier Amplitude Sensitivity Test (FAST) [6–9] is used to derive first-order sensitivity metrics F_i of parameters i on several process outputs of the PRMS model [10]. This is in contrast to the work presented here, which derives the sensitivity of model processes (instead of model parameters)

on the simulated streamflow (instead of model processes). Another major difference with the work by Markstrom *et al.* [5] is that they derive the sensitivities regarding summary statistics (mean, coefficient of variation (CV), and autoregressive lag-1 correlation coefficient (AR- 1)) of these process outputs, rather than deriving the time-dependent sensitivities, as in the work presented herein. Although the setup of the two studies and the target variables are different a consistency check would yield an insight into how transferable the results are, and into whether the work presented herein is an extension and generalization of that previous work.

We therefore derived, based on the saved model outputs of all our runs, the first-order Sobol' sensitivities of the model parameters of the blended model regarding the mean simulated streamflow, i.e., $S_i(\overline{Q(t)})$ rather than the metric that is used throughout the study presented in the main manuscript $S_i(Q(t))$. The $S_i(Q(t))$ might then be aggregated by variance-weighting time steps into $S_i^w(Q(t))$. Since the models are non-linear, major differences are expected between $S_i(\overline{Q(t)})$ and $S_i^w(Q(t))$ even though they are average sensitivities of the model parameters. The sensitivities are derived for the 3316 basins in this study. The basin-wise, sum of first-order Sobol' sensitivity index estimates S for all blended model parameters i regarding the mean simulated streamflow $\overline{Q_t}$ is denoted from here on as $\sum S_i(\overline{Q_t})$.

The FAST analysis by Markstrom *et al.* [5] is based on hydrologic response units (HRUs). The data were shared by Steven Markstrom after personal correspondence. To enable a comparison with the basin-wise estimates above, the mean area-weighted FAST sensitivities per basin are derived. The Markstrom *et al.* [5] study was performed for the continental United States (CONUS), and as a result, around 400 Canadian basins could not be included for comparison. The FAST results are available for 2918 basins within the CONUS domain. Since Markstrom *et al.* [5] derived the parameter sensitivities of model process outputs (rather than streamflow), we used the "runoff" process, which is defined as "[...] the total flow from the HRU contributing to streamflow in the connected stream segment [...]" in the PRMS model. It is thus the process output that is closest to streamflow. The basin-wise, sum of first-order FAST sensitivity index estimates F for all PRMS model parameters i regarding the mean simulated runoff $\overline{R_t}$ (Fig. 2 in Markstrom *et al.* [5]; see panel for "mean runoff") is denoted from here on as $\sum F_i(\overline{R_t})$.

Figure S2 opposes the xSSA-derived basin-wise sum of first-order parameter sensitivities of the blended model $\sum S_i(\overline{Q_t})$ to the FAST-derived basin-wise sum of first-order parameters sensitivities of the PRMS model $\sum F_i(\overline{R_t})$. Even though the parameters, models and output variables differ, the spatial patterns of sensitive versus insensitive regions are surprisingly consistent. The sum of first-order sensitivities basically estimates the degree to which the variability in the model output (here streamflow and runoff) can be explained by the individual parameters when varied independently. The closer the sum is to 1.0, the more additive a model is, while the difference of the sum to 1.0 is an indicator of the presence of interactions in the model [11]. Most of the model output variability can be explained by the sum of the first-order sensitivities in the mountainous regions of the Rocky mountains and the Appalachian ranges (green). The arid regions of the Midwest and the coastal/interior plains (pink) are less sensitive, i.e., less additive. However, the estimates for the blended model (Fig. S2a) are generally closer to 1.0 than for the PRMS model (Fig. S2b), which in turn means the blended model is more additive, with fewer parameter interactions, which is usually regarded to be a beneficial property for models.

However, in 557 of the 3316 analyzed basins, the xSSA-derived sum of first-order Sobol' indexes is larger than 1.0. This is a numerical artifact as the sum of first-order indexes has an upper limit of 1.0, indicating that the sensitivity estimates have not yet converged, and more model runs would be required. This is considered somewhat unimportant here since (a) the sensitivity regarding mean streamflow is not a sensitivity used in this study other than for this comparison, (b) the first-order indexes are of secondary importance in general as the total effect, including the parameter interaction, is known to converge faster [12], and (c) the exceedance of the upper limit of 1.0 is minor in most of the 557 basins (median $\sum S_i(\overline{Q_t}) = 1.022$). The Pearson correlation coefficient between the $\sum S_i(\overline{Q_t})$ and $\sum F_i(\overline{R_t})$ of the 2918 basins that exist in both analyses is 0.8770 when the xSSA estimates are truncated at 1.0 and 0.7552 if the values are not truncated.

The surprisingly close agreement between the two results indicates that the xSSA analysis that derives the sensitivity of model parameters and model processes on streamflow is indeed consistent with the previous large-scale continental study of parameter sensitivities on hydrologic processes, and can therefore be regarded as an extension of previous knowledge.

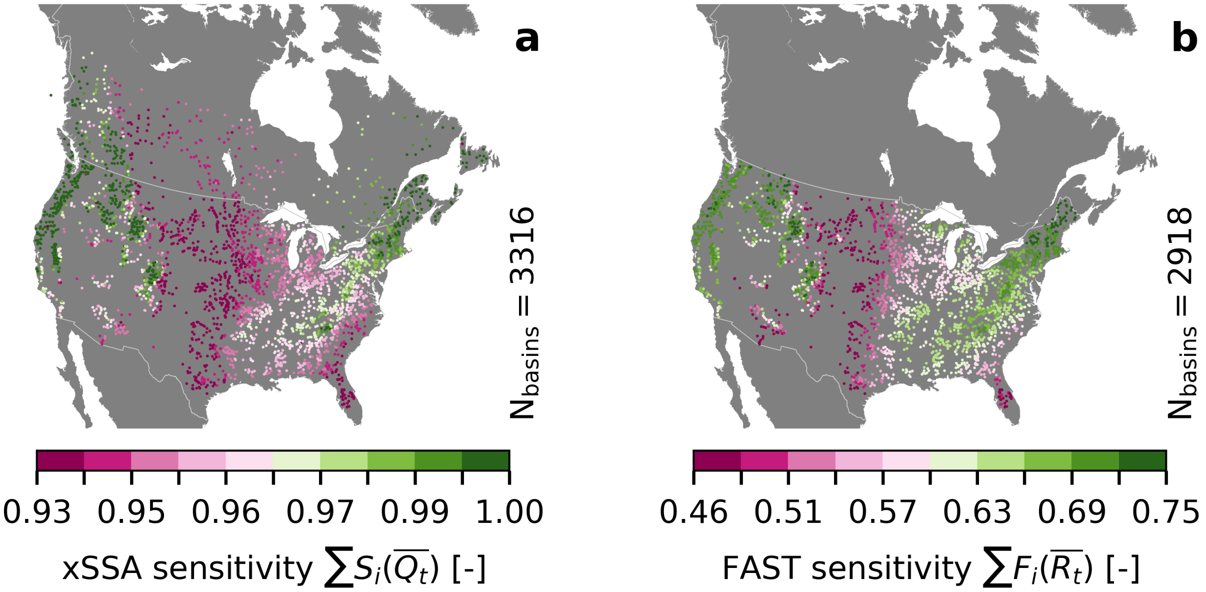

FIG. S2. **Comparison of xSSA sensitivity results with previous continental study.** The (a) basin-wise, sum of first-order Sobol' sensitivity index estimates S for all blended model parameters i regarding the mean simulated streamflow $\overline{Q_t}$ are contrasted to the (b) basin-wise, sum of first-order FAST sensitivity index estimates F for all PRMS model parameters i regarding the mean simulated runoff $\overline{R_t}$ derived by Markstrom *et al.* [5]. Sensitivity estimates close to 1.0 in both analyses indicate additive models with almost no presence of interactions in the model. The xSSA analysis was applied to 3316 basins in North America, while the FAST analysis was performed over the continental US. The HRU-based estimates of the latter study were aggregated to basin-wise estimates to allow a comparison.

REFERENCES

* E-mail corresponding author at: juliane.mai@uwaterloo.ca

[1] J. R. Craig, G. Brown, R. Chlumsky, R. W. Jenkinson, G. Jost, K. Lee, J. Mai, M. Serrer, N. Sgro, M. Shafii, A. P. Snowdon, and B. A. Tolson, Flexible watershed simulation with the Raven hydrological modelling framework, Environmental Modelling & Software **129**, 104728 (2020).

[2] J. Mai, J. R. Craig, and B. A. Tolson, Simultaneously determining global sensitivities of model parameters and model structure, Hydrology and Earth System Sciences **24**, 5835 (2020).

[3] R. Chlumsky, J. Mai, J. R. Craig, and B. A. Tolson, Simultaneous Calibration of Hydrologic Model Structure and Parameters Using a Blended Model, Water Resources Research **57**, e2020WR029229 (2021).

[4] J.-L. Martel, K. Demeester, F. Brissette, A. Poulin, and R. Arsenault, HMETS—A Simple and Efficient Hydrology Model for Teaching Hydrological Modelling, Flow Forecasting and Climate Change Impacts, International Journal of Engineering Education **33**, 1307 (2017).

[5] S. L. Markstrom, L. E. Hay, and M. P. Clark, Towards simplification of hydrologic modeling: identification of dominant processes, Hydrology and Earth System Sciences **20**, 4655 (2016).

[6] J. H. Schaibly and K. E. Shuler, Study of the sensitivity of coupled reaction systems to uncertainties in rate coefficients. II Applications, Journal of Chemical Physics **59**, 3879 (1973).

[7] R. I. Cukier, C. M. Fortuin, K. E. Shuler, A. G. Petschek, and J. H. Schaibly, Study of the sensitivity of coupled reaction systems to uncertainties in rate coefficients. I Theory, Journal of Chemical Physics **59**, 3873 (1973).

[8] R. I. Cukier, J. H. Schaibly, and K. E. Shuler, Study of the sensitivity of coupled reaction systems to uncertainties in rate coefficients. III. Analysis of the approximations, Journal of Chemical Physics **63**, 1140 (1975).

[9] A. Saltelli, M. Ratto, S. Tarantola, and F. Campolongo, Sensitivity analysis practices: Strategies for model-based inference, Reliability Engineering & System Safety **91**, 1109 (2006).

[10] S. L. Markstrom, R. S. Regan, L. E. Hay, R. J. Viger, R. M. Webb, R. A. Payn, and J. H. LaFontaine, PRMS-IV, the Precipitation-Runoff Modeling System, Version 4., in *U.S. Geological Survey Techniques and Methods, book 6, chapt. B7* (U.S. Department of the Interior, U.S. Geological Survey, 2015) p. 158.

[11] A. Saltelli, M. Ratto, T. Andres, F. Campolongo, J. Cariboni, D. Gatelli, M. Saisana, and S. Tarantola, *Global Sensitivity*

Analysis, The Primer (Wiley-Interscience, 2008).
[12] M. Cuntz, J. Mai, M. Zink, S. Thober, R. Kumar, D. Schäfer, M. Schrön, J. Craven, O. Rakovec, D. Spieler, V. Prykhodko, G. Dalmasso, J. Musuuza, B. Langenberg, S. Attinger, and L. Samaniego, Computationally inexpensive identification of noninformative model parameters by sequential screening, Water Resources Research **51**, 6417 (2015).
